# Emerging Targets and Treatments for Sarcopenia: A Narrative Review

**DOI:** 10.3390/nu16193271

**Published:** 2024-09-27

**Authors:** Stefano Cacciatore, Riccardo Calvani, Ilaria Esposito, Claudia Massaro, Giordana Gava, Anna Picca, Matteo Tosato, Emanuele Marzetti, Francesco Landi

**Affiliations:** 1 Department of Geriatrics, Orthopedics and Rheumatology, Università Cattolica del Sacro Cuore, L.go F. Vito 1, 00168 Rome, Italy; riccardo.calvani@unicatt.it (R.C.); ilaria.esposito1@unicatt.it (I.E.); claudia.massaro02@icatt.it (C.M.); giordana.gava01@icatt.it (G.G.); francesco.landi@unicatt.it (F.L.); 2 Fondazione Policlinico Universitario “Agostino Gemelli” IRCCS, L.go A. Gemelli 8, 00168 Rome, Italy; picca@lum.it (A.P.); matteo.tosato@policlinicogemelli.it (M.T.); 3 Department of Medicine and Surgery, LUM University, Strada Statale 100 Km 18, 70100 Casamassima, Italy

**Keywords:** physical performance, muscle health, satellite cells, mitochondrial dysfunction, hydration, neuromuscular junction, inflammaging

## Abstract

Background: Sarcopenia is characterized by the progressive loss of skeletal muscle mass, strength, and function, significantly impacting overall health and quality of life in older adults. This narrative review explores emerging targets and potential treatments for sarcopenia, aiming to provide a comprehensive overview of current and prospective interventions. Methods: The review synthesizes current literature on sarcopenia treatment, focusing on recent advancements in muscle regeneration, mitochondrial function, nutritional strategies, and the muscle–microbiome axis. Additionally, pharmacological and lifestyle interventions targeting anabolic resistance and neuromuscular junction integrity are discussed. Results: Resistance training and adequate protein intake remain the cornerstone of sarcopenia management. Emerging strategies include targeting muscle regeneration through myosatellite cell activation, signaling pathways, and chronic inflammation control. Gene editing, stem cell therapy, and microRNA modulation show promise in enhancing muscle repair. Addressing mitochondrial dysfunction through interventions aimed at improving biogenesis, ATP production, and reducing oxidative stress is also highlighted. Nutritional strategies such as leucine supplementation and anti-inflammatory nutrients, along with dietary modifications and probiotics targeting the muscle–microbiome interplay, are discussed as potential treatment options. Hydration and muscle–water balance are emphasized as critical in maintaining muscle health in older adults. Conclusions: A combination of resistance training, nutrition, and emerging therapeutic interventions holds potential to significantly improve muscle function and overall health in the aging population. This review provides a detailed exploration of both established and novel approaches for the prevention and management of sarcopenia, highlighting the need for further research to optimize these strategies.

## 1. Introduction

**Figure 1:**
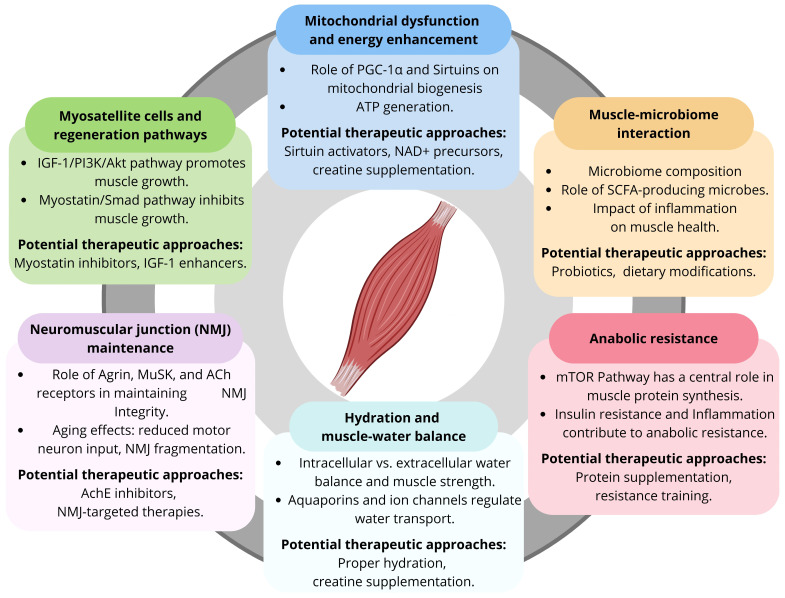
Overview of emerging pathways and potential therapeutic approaches for age-related sarcopenia. Abbreviations: Ach, acetylcholine; AchE, acetylcholinesterase; Akt, protein kinase B; ATP, adenosine triphosphate; IGF-1, insulin-like growth factor 1; mTOR: mechanistic target of rapamycin; MuSK, muscle-specific kinase; NAD+, nicotinamide adenine dinucleotide; NMJ, neuromuscular junction; PGC-1α, peroxisome proliferator-activated receptor gamma coactivator 1-alpha; PI3K, phosphoinositide 3-kinase; SCFA, short-chain fatty acids.

Sarcopenia is a progressive and generalized skeletal muscle disease characterized by accelerated loss of muscle mass, strength, and physical function [1]. While its essential components and operational definitions are still under debate [2], it has been clarified that sarcopenia is not part of normal aging, rather a distinct condition that requires clinical attention and intervention [3,4]. Sarcopenia increases the risk of physical disability and impacts the quality of life, independence, and overall survival of affected individuals [5,6,7]. To date, the global prevalence rates of sarcopenia and severe sarcopenia in adults over the age of 60 are 8–36% and 2–9%, respectively [8]. However, these percentages are expected to rise in the coming years, making it a major public health concern [9]. Currently, pharmacological interventions for sarcopenia remain limited [10], and therapeutic approaches are mainly based on nutritional interventions and physical exercise [11,12]. However, recent developments in the understanding of the pathophysiological pathways of sarcopenia have led to the discovery of specific biomarkers and possible targets for its prevention and treatment (Figure 1) [13,14]. The aim of this narrative review is to provide a comprehensive appraisal of emerging targets and potential treatments for sarcopenia. After discussing the current evidence across various aspects of sarcopenia treatment, each section provides a summary of findings along with current clinical practices and recommendations for managing sarcopenia.

## 2. Literature Search

This narrative review was conducted through a comprehensive search of peer-reviewed literature on sarcopenia and its emerging treatments. Major databases, including PubMed, Scopus, and Web of Science, were used to identify relevant studies published up to 31 August 2024. The search strategy focused on broad keywords such as “sarcopenia”, “muscle regeneration”, “mitochondrial dysfunction”, “muscle-water balance”, “muscle-microbiota interplay”, “nutrient flow”, “anabolic resistance”, and “neuromuscular junction”, or related keywords. As a narrative review, this article aimed to provide a broad overview rather than a systematic synthesis of the evidence, and priority was given to randomized controlled trials, meta-analyses, systematic reviews, and high-impact studies. Inclusion criteria were (1) articles published in English and (2) studies involving human subjects, particularly older adults. Exclusion criteria were (1) articles with unclear methodologies or insufficient clinical data, or (2) preclinical studies without clear translational potential. The review was further refined by cross-referencing citations from included articles to ensure comprehensive coverage of the most recent advancements in the field.

## 3. Muscle Regeneration in Sarcopenia

Muscle regeneration is a critical process in maintaining muscle mass and function, and it is often impaired in sarcopenia [15,16]. Muscle regeneration involves the activation, proliferation, and differentiation of myosatellite cells (MSCs), which are specialized stem cells of the muscle tissue. Upon injury or stress, MSCs are activated and proliferate to repair and regenerate damaged muscle fibers. Several signaling pathways are involved in regulating MSC activity and muscle regeneration. For instance, insulin-like growth factor-1 (IGF-1) has been shown to stimulate MSC proliferation and differentiation through the activation of the phosphoinositide 3-kinase (PI3K)/Akt pathway, which promotes protein synthesis and inhibits apoptosis [17]. Conversely, myostatin, a member of the transforming growth factor-beta (TGF-β) superfamily, acts as a negative regulator of muscle growth by inhibiting satellite cell activation and proliferation [17].

Research has focused on understanding the relationship between these signaling pathways and their possible therapeutic applications. Targeting myostatin signaling emerged as a promising strategy to enhance muscle regeneration [18]. Monoclonal antibodies and receptor antagonists targeting the myostatin signaling pathway have been shown to increase muscle mass and strength in preclinical models [19]. Such molecules may act as direct inhibitors of myostatin, inhibitors of myostatin activation, and blockers of the receptors involved in myostatin signaling [20]. Bimagrumab (Novartis/Versanis) acts as an agonist by binding to activin type 2 receptors and preventing ligands from binding and activating these receptors. By blocking the binding site of the activin type 2 receptor, it increases circulating levels of ligands (e.g., bone morphogenetic protein (BMP)-9 and BMP-10) that can bind to other receptors, such as BMP receptor 2, thereby further promoting muscle growth and counteracting the effects of myostatin/activin A signaling [20]. Clinical trials are ongoing to evaluate the efficacy and safety of myostatin inhibitors in older adults with sarcopenia; however, preliminary results indicate inconsistent benefits in improving muscle function and reducing the occurrence of physical frailty and disability [21,22,23,24,25,26].

Another area of research focuses on the role of chronic inflammation in the impairment of MSC activity and muscle regeneration [27]. The suppression of MSC proliferation and differentiation has been linked to the action of proinflammatory cytokines, such as tumor necrosis factor-alpha (TNF-α) and interleukin-6 (IL-6) [28,29]. Conversely, anti-inflammatory therapies, including the use of cytokine inhibitors and non-steroidal anti-inflammatory drugs (NSAIDs), have shown potential in enhancing muscle regeneration and reducing muscle loss associated with sarcopenia [30,31]. However, significant side-effects of NSAIDs in older adults, such as gastrointestinal and cardiovascular complications, pose a challenge for their widespread use in clinical practice [32].

Together with MSCs, the extracellular matrix (ECM) is an important component in muscle regeneration. The ECM provides structural support and regulates the availability of growth factors and signaling molecules necessary for MSC function. Remodeling of the ECM is essential for efficient muscle repair, and a dysregulation of ECM components has been linked to impaired regeneration in sarcopenia [33,34]. Preliminary results indicate that therapeutic strategies aimed at modulating ECM composition and function, such as the use of matrix metalloproteinase inhibitors, may be beneficial to enhance muscle regeneration [35,36]; however, further studies are required in this field.

Another area of active investigation involves the role of the MSC niche in muscle regeneration. The niche, comprising various cell types and extracellular components surrounding MSCs, provides critical signals that regulate MSC behavior. Alterations in the niche, such as changes in cell composition or ECM structure, can impact MSCs and muscle regeneration [37,38]. Strategies aimed at restoring the niche environment, such as cell-based therapies or bioengineered scaffolds, are being explored to improve muscle regeneration in the setting of sarcopenia [39,40,41]. However, they are still in early stages of research, and further studies are required to determine their efficacy and safety in clinical settings.

Advances in gene editing technologies, particularly CRISPR/Cas9, have opened new possibilities for muscle regeneration therapies. Gene editing can be used to correct genetic mutations that impair muscle function or to enhance the expression of genes involved in muscle growth and regeneration. For instance, CRISPR/Cas9-mediated knockout of myostatin has been demonstrated to increase muscle mass and strength in animal models [42]. Translating these findings to clinical applications holds promise for developing novel treatments for sarcopenia and other muscle-wasting conditions, with a possible role of RNA-based gene therapy in the coming decades [43]. While preclinical findings are promising, translating these technologies to clinical applications remains challenging and raises ethical considerations.

Stem cell therapy is another promising approach for enhancing muscle regeneration leveraging on stem cell populations other than MSCs [43]. Mesenchymal stem cells can differentiate into myogenic cells and secrete paracrine factors that promote MSC activity and muscle repair [44]. Induced pluripotent stem cells derived from reprogrammed somatic cells offer the advantage of generating patient-specific stem cells for autologous transplantation, reducing the risk of immune rejection [45]. Recent clinical trials have shown promising results [46,47]; however, further studies are needed to evaluate the safety and efficacy of leveraging stem cells for sarcopenia and physical frailty in humans.

Emerging research is also exploring the role of microRNAs (miRNAs) in regulating muscle regeneration. miRNAs are small non-coding RNAs that modulate gene expression by targeting messenger RNAs for degradation or translational repression. Several miRNAs have been identified as key regulators of MSC function and muscle regeneration. For instance, miR-1 and miR-206 promote myogenic differentiation, while miR-489 maintains satellite cell quiescence [48]. Therapeutic modulation of miRNA expression, using miRNA mimics or inhibitors, offers a novel approach to enhance muscle regeneration and treat sarcopenia [48]; yet, extensive research is needed to explore its potential clinical applications.

More conventional approaches that rely on diet (Table 1) and physical activity (Table 2) have been shown to promote muscle regeneration.

Adequate protein intake is essential for providing the building blocks necessary for muscle repair and growth [11,49], while a balanced diet rich in antioxidants and anti-inflammatory nutrients can help reduce inflammation and promote muscle rejuvenation [52,64]. Specific amino acids, such as leucine, have been shown to stimulate muscle protein synthesis (MPS) and enhance MSC activity [49,50,65]. β-hydroxy-β-methyl butyrate (HMB) is a metabolite of the amino acid leucine, recognized for its role in promoting muscle health. HMB modulates chronic inflammation by reducing the levels of pro-inflammatory cytokines and inhibiting the nuclear factor kappa-light-chain-enhancer of the activated B cells (NF-κB) signaling pathway, which is crucial in the inflammatory response [54]. Moreover, HMB enhances the proliferation and differentiation of MSCs [54]. The anti-inflammatory and pro-regeneration effects of HMB are particularly beneficial in conditions of muscle wasting and chronic diseases [55]. In addition, nutritional supplements, including whey protein, creatine and omega-3 fatty acids, have been studied for their potential to support muscle recovery and counteract sarcopenia [66]. Exercise induces mechanical stress on muscle fibers, which triggers the activation of various signaling pathways involved in muscle regeneration. Resistance training, in particular, has been shown to activate MSCs, increase MPS, and enhance muscle strength and mass [67]. Combining exercise with other therapeutic interventions, such as nutritional supplementation or pharmacological agents, may provide synergistic benefits in promoting muscle regeneration and improving functional outcomes in sarcopenia [68]. Adherence to exercise programs, however, can be challenging, especially for older individuals with limited mobility or other health conditions. The optimal combination of exercise with nutritional or pharmacological interventions also requires further investigation to identify the most effective approaches for different patient populations. Additionally, while exercise is a powerful tool for combating sarcopenia, it may not be sufficient for severe sarcopenia, which might require additional interventions.

**Table 2:** Exercise interventions and outcomes in sarcopenia.

Exercise Intervention	Types of Exercise	Effects on Muscle Mass	Effects on Muscle Strength	Effects on Muscle Function	Synergistic Effects with Other Therapies	References
Resistance training	Weightlifting, resistance bands, bodyweight exercises	Increases muscle mass	Increases muscle strength, especially in large muscle groups	Enhances functional performance in daily activities (e.g., walking, stair climbing)	Synergistic with protein supplementation and HMB in promoting muscle growth and strength	[67,68]
Aerobic exercise	Walking, cycling, swimming	May slightly increase muscle mass	Improves muscle endurance, with minor strength gains	Enhances cardiovascular fitness and overall functional capacity	May complement resistance training by improving mitochondrial function	[69,70]
Combined resistance and aerobic training	Alternating or concurrent resistance and aerobic workouts	Increases muscle mass, potentially more than aerobic exercise alone	Improves both strength and endurance	Enhances both muscular and cardiovascular function, leading to improved overall fitness	Synergistic with dietary interventions such as protein or creatine supplementation for comprehensive muscle health benefits	[70,71]
High-intensity interval training	Short bursts of intense exercise followed by rest	May increase muscle mass, especially in untrained individuals	Increases strength and endurance, depending on the intensity	Enhances functional capacity, especially in tasks requiring both power and endurance	Synergistic with creatine supplementation for enhancing both strength and recovery	[72]
Flexibility and balance training	Yoga, tai chi, stretching	Marginal direct effects on muscle mass	May improve strength through enhanced muscle control	Improves balance, reducing the risk of falls in older adults	Complements other exercise modalities by improving mobility and reducing injury risk during resistance training	[73]

Abbreviation: HMB, β-hydroxy β-methylbutyrate.

### Summary of Evidence and Current Clinical Recommendations

Muscle regeneration relies on the activation and proper function of MSCs, which are key to muscle repair. The IGF-1/PI3K/Akt and myostatin pathways regulate MSC activity. Although targeting myostatin has shown promise in preclinical models, clinical results are inconsistent. Chronic inflammation also impairs MSC function and, while anti-inflammatory therapies have potential, their use is limited by side-effects. Emerging treatments, like gene editing and stem cell therapy, hold promise but are still experimental.

Resistance training and adequate protein intake remain the primary interventions for supporting muscle regeneration in older adults. Myostatin inhibitors and anti-inflammatory therapies are not yet recommended due to inconsistent efficacy and potential risks. Future therapies involving stem cells and gene editing require more research before being applied in clinical settings.

## 4. Mitochondrial Dysfunction and Energy Enhancers in Sarcopenia

Mitochondrial dysfunction is one of the hallmarks of aging [74] and one of the main drivers in the development of age-related sarcopenia [75]. In the setting of sarcopenia, mitochondrial failure, mostly involving the ability to replace defective mitochondria, decreased adenosine triphosphate (ATP) generation, and increased oxidative stress, is a significant contributor to muscle weakness and fatigue [76]. The aging process itself induces several changes in mitochondrial quality control, including a decrease in mitochondrial biogenesis, alterations in mitochondrial DNA, and an increase in the production of reactive oxygen species [77]. These changes impair the ability of muscle cells to generate and sustain energy, highlighting the critical need for interventions that enhance mitochondrial function and energy metabolism.

Potential targets for energy enhancement in the management of sarcopenia focus on improving mitochondrial biogenesis, ATP production, and overall mitochondrial quality control. The peroxisome proliferator-activated receptor gamma coactivator 1-alpha (PGC-1α), a key regulator of mitochondrial biogenesis, enhances the production of new mitochondria, improves mitochondrial function, and increases oxidative capacity [78]. Similarly, sirtuins, NAD^+^-dependent deacetylases like SIRT1 and SIRT3, regulate mitochondrial biogenesis and function, and enhancing their activity through pharmacological agents or calorie restriction mimetics holds promise for improving muscle energy metabolism [79]. Pharmacological sirtuin activators, such as resveratrol and NAD^+^ precursors like nicotinamide riboside, have demonstrated potential in enhancing mitochondrial function and reducing oxidative stress [49]. However, robust clinical evidence supporting the efficacy of these interventions in older adults with sarcopenia remains lacking. Many of these compounds are still in early stages of research, and their long-term safety and efficacy need further validation before they can be recommended in clinical practice.

ATP production and utilization can be ameliorated by creatine monohydrate, an amino acid derivative that facilitates rapid ATP production by donating a phosphate group to adenosine diphosphate, particularly beneficial in short-duration, high-intensity activities [56]. Adenosine monophosphate-activated protein kinase (AMPK), a cellular energy sensor, maintains energy homeostasis by enhancing glucose uptake, fatty acid oxidation, and mitochondrial biogenesis, with pharmacological activators like metformin and 5-aminoimidazole-4-carboxamide ribonucleotide (AICAR) being explored for their potential to enhance muscle energy metabolism and combat sarcopenia [69,80,81]. Despite these benefits, creatine and AMPK activators are not effective for all older adults. The individual variability in response to these supplements, as well as potential side-effects (such as gastrointestinal discomfort from creatine or the risk of hypoglycemia with metformin), limits their widespread application. Additionally, further clinical trials are needed to confirm their long-term benefits specifically in older adults with sarcopenia.

Mitochondrial quality control can be improved through mitophagy, the selective autophagic degradation of damaged mitochondria, which may be promoted via pharmacological agents or lifestyle interventions (e.g., exercise) [82]. Additionally, antioxidants like coenzyme Q10 (CoQ10), a key component of the mitochondrial electron transport chain, and N-acetylcysteine (NAC) mitigate oxidative damage, preserve mitochondrial integrity, and improve energy production [61,83]. However, clinical data supporting the use of antioxidants in sarcopenia are not robust, and more rigorous trials are necessary to establish their long-term efficacy. Additionally, while mitophagy is a theoretical target for enhancing mitochondrial quality, practical interventions that effectively enhance mitophagy in humans are still in the experimental phase.

l-carnitine, essential for transporting long-chain fatty acids into mitochondria for β-oxidation, improves muscle function, reduces fatigue, and boosts exercise performance [84]. Branched-chain amino acids (BCAAs), particularly leucine, stimulate MPS, improve muscle mass, and reduce muscle breakdown, while also serving as an energy source during prolonged exercise [51]. l-carnitine and BCAAs have demonstrated beneficial effects in terms of reducing muscle fatigue and enhancing muscle recovery, particularly when used in combination with exercise interventions. Notwithstanding these findings, more studies are needed to better understand the optimal dosages and long-term impacts of these supplements.

Regular exercise, especially resistance and endurance training, promotes mitochondrial biogenesis, improves oxidative capacity, and enhances insulin sensitivity, contributing to better muscle function and overall health [70]. Hormonal therapies, including testosterone, growth hormone, and IGF-1, have shown potential in improving muscle mass, strength, and function [85]. Yet, the use of hormonal therapies requires careful consideration of risks and benefits. Hormone replacement can lead to serious side-effects, including cardiovascular events and increased cancer risk.

### Summary of Evidence and Current Clinical Recommendations

Mitochondrial dysfunction, characterized by reduced ATP production and increased oxidative stress, is considered to be a key factor in muscle weakness in sarcopenia. Enhancing mitochondrial biogenesis and improving mitochondrial quality through pathways like PGC-1α and sirtuins can potentially improve muscle energy metabolism. Pharmacological activators of sirtuins and AMPK, along with supplements such as creatine and CoQ10, have shown promise in early research but lack robust clinical data.

Physical exercise, particularly resistance and aerobic training, remains the most effective intervention for addressing mitochondrial dysfunction in older adults. Supplements like creatine and CoQ10 may offer additional benefits but require further validation. Pharmacological agents targeting mitochondrial function are still under development and not recommended in clinical practice.

## 5. Hydration and Muscle–Water Balance in Sarcopenia

Hydration is essential in cellular processes, nutrient transport, and biochemical reactions within muscle tissue, and both intracellular (ICW) and extracellular water (ECW) content play significant roles in muscle function and health [86]. ICW, which constitutes a large portion of muscle mass, is essential for maintaining cellular homeostasis, protein synthesis, and metabolic functions. Conversely, ECW, which is primarily found in the interstitial spaces and blood, supports nutrient transport and waste removal. The balance between these two compartments is finely regulated by various molecular mechanisms involving aquaporins, ion channels, and transporters [87]. Dysregulation of this balance can lead to muscle atrophy, impaired muscle contractility, and overall muscle weakness, all of which are hallmark features of sarcopenia [86,88,89,90].

ICW content in lean mass has been proposed as a marker of muscle quality and cellular hydration. A growing body of evidence suggest that ICW is associated with strength, physical function, and frailty [86]. During aging, a relative expansion of ECW compared to ICW accompanies the reduction in muscle mass [91]. The loss of ICW is associated with activation of catabolic processes and concomitant inhibition of the mechanistic target of rapamycin complex 1 (mTORC1) and insulin-stimulated anabolic pathways [92]. In a cohort of community-dwelling individuals aged 65–90 years, the ratio of ECW to ICW in the thigh was found to be negatively associated with knee extension strength and walking speed [93]. This finding suggests that cellular hydration may play a protective role against weakness and functional decline. However, it is possible that the observed reduction in ICW with aging is primarily due to a decrease in muscle mass, raising the question of whether the beneficial effects of higher ICW should be attributed to a greater muscle quantity or enhanced muscle quality through improved cellular hydration. The same researchers proposed a new indicator of muscle quality, defined as ICW content per unit of lean mass (mL/kg). This ratio was observed to decrease with age and to be positively correlation with muscle strength and function, and negatively related with frailty risk, irrespective of age, sex, and the number of diseases [86,89].

ICW content is closely linked to muscle cell volume and function. Aquaporins, a family of water channel proteins, facilitate the transport of water across cell membranes and are crucial for maintaining intracellular hydration. Among them, aquaporin-4 (AQP4) is predominantly expressed in skeletal muscle and plays a significant role in muscle physiology. Changes in AQP4 expression or function can disrupt ICW homeostasis, leading to muscle fiber swelling or shrinkage, which subsequently affects muscle strength and endurance [94]. Ion channels, such as sodium-potassium pumps (Na+/K+ ATPase) and chloride channels, also contribute to water movement by creating osmotic gradients that drive water into or out muscle cells [95]. Dysfunctions in these ion channels can alter intracellular osmolality, thereby impacting muscle cell hydration and function [96].

ECW balance is equally vital for muscle health. It ensures the proper delivery of oxygen and nutrients to muscle fibers and the removal of metabolic byproducts. The ECM, composed of collagen, elastin, and proteoglycans, is essential for regulating ECW content and preserving muscle tissue structure. Proteoglycans, in particular, have a high affinity for water and contribute to the viscoelastic properties of the ECM. In the setting of sarcopenia, changes in ECM composition and function can lead to increased ECW retention, edema, and inflammation, exacerbating muscle degeneration [34]. However, changes in ECM composition that contribute to ECW retention and inflammation are difficult to reverse, particularly in older adults with advanced sarcopenia. While hydration strategies may be useful, they may not fully counteract the detrimental effects of ECM dysregulation.

Several molecular mechanisms underpin the regulation of muscle–water balance. The mTORC signaling pathway is a key regulator of muscle cell growth and protein synthesis, and it also influences cellular hydration status. mTORC activity is sensitive to changes in cellular hydration, with hypo-osmotic stress (cell swelling) activating mTORC signaling, while hyper-osmotic stress (cell shrinkage) inhibits it. This dynamic response helps coordinate protein synthesis with cellular hydration state, ensuring optimal muscle function [82].

Maintaining proper hydration is essential for muscle function and overall health, especially in older adults at risk for sarcopenia. The European guidelines recommend older persons to ingest a daily water intake of 1.6 L for women and 2.0 L for men to maintain optimal hydration [60]. These recommendations aim to counteract the decreased thirst sensation and reduced renal function often observed in older adults, which can lead to insufficient fluid intake and dehydration. To achieve the necessary hydration levels, an integrated approach is required, including lifestyle modifications, dietary modifications, and the use of nutritional supplements to improve water intake and retention. 

Supplementation with electrolytes and nutrients that support cellular hydration can also help maintain muscle–water balance and improve muscle performance. Beyond its role in energy metabolism, creatine helps retain ICW, supporting proper muscle hydration [57]. BCAAs, especially leucine, support MPS and hydration. They can also serve as an energy source during prolonged exercise, reducing muscle fatigue and supporting hydration [97]. Resistance training has been shown to increase muscle mass and strength, partly by enhancing ICW content and promoting favorable changes in muscle cell volume [71,72]. 

Medical interventions, including treatments that modulate aldosterone and antidiuretic hormone (ADH) pathways, may also help manage hydration levels in older adults who struggle to maintain adequate fluid balance [98,99].

### Summary of Evidence and Current Clinical Recommendations

Proper hydration is crucial for muscle function. Aging leads to a reduction in ICW and an increase in ECW, contributing to muscle weakness and functional decline. Dysregulation of muscle–water balance impairs muscle metabolism, and factors such as aquaporin function and mTOR signaling are involved in maintaining hydration status.

Older adults are recommended to maintain adequate hydration, with daily water intake guidelines set at 1.6 L for women and 2.0 L for men. Resistance training and supplements like creatine may help support muscle hydration. Electrolyte supplementation and interventions targeting fluid regulation, such as those affecting aldosterone and ADH pathways, may benefit individuals who have difficulties with maintaining fluid balance.

## 6. Muscle–Microbiota Interplay in Sarcopenia

The human gut harbors a complex community of microorganisms known as the gut microbiota, which maintains a symbiotic and commensal relationship with the host [100]. The gut microbiota plays essential roles in the health of the host. It aids in nutrient absorption, synthesizes essential vitamins, regulates the immune system, and protects against pathogens. Additionally, it contributes to the host’s metabolism, assists in mineral absorption, regulates fat storage, activates bioactive compounds, and serves as an endocrine and neuronal organ [101,102,103]. 

The human gut microbiota undergoes distinct transformations across different life stages, characterized by rapid changes in infancy, stability in adulthood, and shifts in old age [104,105]. Despite advancements, understanding the timing and effects of these age-related changes on host physiology remains challenging. Recent research suggests that alterations in the gut microbiome of centenarians and older individuals may be adaptations to aging rather than deviations from a healthy microbial profile [106], holding clinical significance for diagnostics and personalized microbiota modulation.

Inflammaging, immune senescence, and dietary modifications contribute to changes in gut microbiota composition and function in older adults, especially in frailer individuals [107]. Studies consistently show a decline in microbial stability and diversity, accompanied by an increase in pro-inflammatory microbes [106]. In the ELDERMET study, researchers identified a predominant presence of the genera *Bacteroides*, *Alistipes*, and *Parabacteroides* in individuals aged 65 years and older, in comparison to healthy younger controls [108]. Furthermore, significant differences were observed between community-dwelling older adults and long-term care residents [109]. Also, frail individuals in the ELDERMET study displayed elevated levels of lithocholic acid and deoxycholic acid compared with non-frail counterparts [110]. Ghosh et al. [110] found that individuals over 60 years of age exhibited greater variation in gut microbiota composition than younger individuals. Specifically, the gut microbiota of older individuals tends to have lower levels of *Firmicutes*, including *Clostridium* and *Faecalibacterium*, as well as *Bifidobacterium*. Conversely, there is an increase in *Proteobacteria* and *Streptococcaceae*, which is associated with exacerbations in inflammation, frailty, and multimorbidity [111]. These microbial shifts have been linked to sarcopenia-related muscle loss and functional decline. In contrast, the presence of different microbial clusters may correspond with enhanced cardiovascular and metabolic health, possibly acting as indicators of healthy aging and longevity [112]. Furthermore, age-related changes in the gut microbiota impact its metabolic potential, with increased proteolytic activity and variations in compound degradation, which are most likely driven by lifestyle and environmental exposures [111]. 

Recent studies suggest a possible role of the gut microbiota in the pathophysiology of muscle loss [113,114,115,116]. In a cross-sectional analysis of 728 female twins in a community setting, higher frailty status correlated with decreased levels of anti-inflammatory short-chain fatty acids (SCFAs)-producing microbes like *Faecalibacterium prausnitzii* and an increase in pro-inflammatory *Enterobacteriaceae* [117]. Frailer individuals also exhibited heightened lipopolysaccharide synthesis, indicating an elevated local and systemic pro-inflammatory state [118]. The ELDERMET study found that institutionalization and muscle loss were associated with a dysbiosis, reduced anti-inflammatory microbial metabolites such as butyrate, and elevated levels of circulating pro-inflammatory markers like TNF-α, IL-6, IL-8, and C-reactive protein (CRP) [109]. Pro-inflammatory bacteria, including *Eggerthella lenta*, were found in frail patients, possibly contributing to sarcopenia [117]. Sarcopenic and frail individuals exhibited a similar gut microbial profile, marked by enrichment in *Oscillospira* and *Ruminococcus*, and reductions in members of *Barnesiellaceae* and *Christenellaceae* families [119]. Animal studies support these findings, showing that butyrate administration could help restore lean muscle mass [120]. Conversely, medications like proton pump inhibitors and NSAIDs, commonly used by older adults, were associated with dysbiosis, potentially exacerbating frailty and its complications [111]. While animal studies on butyrate administration are promising, clinical evidence in humans remains limited. The optimal way to increase SCFA production through diet or supplementation is not yet well established, and the long-term effects of manipulating microbiota for muscle health require more extensive trials in older populations.

Research was carried out to investigate treatments, such as diet modifications, prebiotics, and probiotics, aimed at restoring a balanced composition of gut microbiota. The NU-AGE project demonstrated that a Mediterranean diet intervention in older adults led to increased levels of beneficial microbial communities, which was associated with reduced frailty and improved cognitive function, along with decreased inflammatory markers such as CRP and IL-17. Additionally, there was an increase in the circulating levels of SCFAs/branched-chain fatty acids and a decrease in the production of secondary bile acids, p-cresols, ethanol, and carbon dioxide [121]. Similarly, dietary supplementation with galacto-oligosaccharides showed promise in restoring beneficial *Bifidobacterium* spp. levels in prefrail older individuals [122]. Increasing dietary fiber intake is highlighted as a beneficial strategy for preventing age-related decline in skeletal muscle mass and strength [111]. Interventional studies, like one led by Lee et al. [123], observed improvements in handgrip strength and overall functional performance in frail older adults following a minimum of six weeks of continuous *L. plantarum* supplementation. While diet can positively influence gut microbiota, individual responses to dietary changes vary significantly. Moreover, the long-term effects of microbiota-targeted dietary interventions on muscle health are not fully understood. Additional longitudinal studies are necessary to determine the most effective dietary strategies for maintaining muscle mass in older adults. Further research with larger cohorts and diverse microbiome analysis methods is needed to fully understand and utilize gut microbiota modulation for promoting healthy aging and counteracting sarcopenia.

### Summary of Evidence and Current Clinical Recommendations

The gut microbiota impacts muscle health by influencing nutrient absorption, inflammation, and metabolism. In older adults, changes in gut microbiota composition, often marked by a reduction in beneficial bacteria and an increase in pro-inflammatory microbes, contribute to the onset of sarcopenia and frailty.

Dietary measures, particularly the Mediterranean diet and an increased fiber intake, as part of multimodal interventions, improve muscle function and reduce the risk of frailty. Prebiotic and probiotic supplementation may offer additional benefits, but further research is needed to confirm their effectiveness in targeting muscle loss.

## 7. Nutrient Flow in Sarcopenia

Nutrient flow refers to the delivery and utilization of essential nutrients that support muscle health, including proteins, amino acids, vitamins, and minerals. A proper nutrient flow is crucial for maintaining muscle protein synthesis (MPS) and preventing muscle protein breakdown (MPB) [124]. Disruptions in nutrient flow, whether due to inadequate dietary intake, impaired digestion, or altered nutrient metabolism and delivery contribute to the pathogenesis of sarcopenia [125,126].

Endothelial cells and smooth muscle cells in microvascular units adapt to the metabolic demands of muscle fibers in response to insulin [127,128]. The muscle microvascular system undergoes structural and functional adaptations essential for regulating nutrient delivery and trans-endothelial transport processes, sustaining muscle metabolism [129]. Studies have demonstrated the critical role of the microvascular system in efficient nutrient delivery to muscles, highlighting the importance of maintaining vascular health for muscle regeneration. Metabolic–vascular coupling in skeletal muscle dynamically coordinates blood flow with metabolic demands, especially during muscle contraction [130]. Blood vessel dilation in response to increased metabolism ensures efficient oxygen and nutrient delivery and waste removal, supporting muscle function and performance [130]. During exercise, the transition from anaerobic to aerobic metabolism requires increased oxygen and nutrient supply, which are facilitated by enhanced blood flow to active muscle tissues [131]. Despite this knowledge, the long-term effects of impaired nutrient flow on muscle health in sarcopenia are not fully understood. Older individuals with cardiovascular disease or multimorbidity may show severe microvascular involvement. Therefore, interventions targeting the microvascular system have yet to demonstrate consistent efficacy in older adults with sarcopenia.

Studies have also explored the role of hormones like glucagon-like peptide 1 (GLP-1) and insulin in regulating the muscle microvascular system and nutrient delivery [132]. GLP-1 and insulin increase muscle endothelial surface area and promote the delivery of oxygen, nutrients, and hormones to muscle tissues, thereby optimizing nutrient transport for muscle function [132]. Regulating blood flow volume may help modulate anabolic resistance in muscle, emphasizing the importance of adequate blood flow for supporting muscle protein synthesis [133]. Age-related changes in muscle blood flow and nutrient transport can affect muscle protein metabolism and anabolic responses to exercise [134]. Impaired microcirculatory blood flow can contribute to anabolic resistance by limiting the delivery of essential amino acids to skeletal muscle, highlighting the importance of optimizing blood flow for muscle health [134]. Adequate blood flow is crucial for modulating MPS and MPB, as it facilitates the transport of metabolic precursors and products, underscoring its role in supporting muscle metabolism [135]. The ability of hormones like insulin and GLP-1 to regulate nutrient delivery and improve endothelial function offers a promising therapeutic pathway for combating sarcopenia. However, the effects of these hormones can be diminished in older adults, particularly in those with insulin resistance, making the benefits of hormone-based strategies less reliable in these populations.

Protein intake and their digestion into amino acids are vital for MPS. Essential amino acids, especially leucine, stimulate the mTORC signaling pathway, a key regulator of MPS. In sarcopenic individuals, the efficiency of this pathway can be compromised, leading to an imbalance between MPS and MPB [124]. Ensuring an adequate intake of high-quality protein, rich in essential amino acids, is thus a key strategy for combating sarcopenia. Studies have shown that older adults often have reduced anabolic responses to protein ingestion, a phenomenon known as anabolic resistance [136]. This resistance can be mitigated by consuming higher quantities of protein or by distributing protein intake evenly across meals to optimize MPS throughout the day [137]. Studies have consistently shown that adequate protein intake, particularly when rich in leucine, can significantly improve MPS and combat muscle loss. However, the effectiveness of protein supplementation is often limited by the degree of anabolic resistance in older adults, necessitating further research to optimize dosing strategies and determine the most effective approaches for mitigating MPB.

The molecular mechanisms underlying nutrient flow and its impact on sarcopenia are complex and involve multiple signaling pathways. The mTORC pathway, as previously mentioned, is central to the regulation of muscle growth and atrophy. In addition to mTORC, the AMPK pathway, which is activated under conditions of low energy availability, can inhibit MPS and promote MPB [138]. IGF-1 is another critical regulator that promotes muscle growth by activating the mTORC pathway and inhibiting the Forkhead box O (FoxO) transcription factors that drive muscle atrophy [139]. Nutrient deficiencies or imbalances can disrupt these signaling pathways, exacerbating muscle loss in sarcopenia. The mTORC and AMPK pathways offer key therapeutic targets for regulating muscle metabolism and promoting muscle health. Nevertheless, interventions that aim to modulate these pathways are still experimental and require more extensive clinical validation before they can be applied to older adults with sarcopenia.

Given the centrality of nutrient flow in the pathogenesis of sarcopenia, targeted nutritional interventions are a promising therapeutic approach. Potential approaches include enhancing protein intake, optimizing the intake of key micronutrients, and incorporating bioactive compounds that can modulate muscle metabolism. For instance, leucine supplementation has been shown to directly stimulate MPS [65,140], while HMB can reduce MPB [54]. Combined supplementation of protein and omega-3 fatty acids has been found to have synergistic effects on muscle health [53]. Some nitrate-rich foods and amino acids, such as arginine and beetroot juice, have vasodilatory properties through their action on the synthesis and/or bioavailability of nitric oxide (NO) [58,62]. Arginine serves as the primary substrate for endothelial NO synthase and is the key precursor for NO production within the vascular endothelium [58]. The consumption of nitrate-rich foods, such as beetroot juice, has been shown to enhance the bioavailability of NO through an alternative nitrate-nitrite-NO pathway, where nitrate is converted to nitrite by commensal bacteria in the oral cavity [63]. By elevating circulating NO levels, beetroot juice and arginine modulate mitochondrial respiration, increase the efficiency of oxygen use during exercise, improve muscle contractility, and enhance blood and nutrient supply to muscle [58,141]. Arginine has also been found to activate mTORC1 by binding to the cytosolic arginine sensor for the mTORC1 subunit 1 (CASTOR1) [59], thus stimulating MPS [142]. Beetroot juice is rich in biologically active substances with anti-inflammatory and antioxidant properties, including betalains, carotenoids, organic acids, and polyphenols, which have beneficial effects for muscle and vessel health [143]. Indeed, the consumption of beetroot juice has been demonstrated to improve endothelial function and tolerance of exercise in older individuals [144]. Nutritional strategies, such as the use of leucine, HMB, and beetroot juice, show promise in improving nutrient flow, muscle metabolism, and overall muscle health. These strategies offer non-invasive, low-risk interventions that may help mitigate muscle loss. However, the evidence for long-term benefits of these interventions in older adults is limited, and further research is needed to confirm their efficacy, particularly in clinical settings.

Exercise has been shown to promote skeletal muscle microvascular blood flow, improving nutrient transport and disposal in muscle tissues [145]. In contrast, high-glucose mixed-nutrient meals have been observed to restrict muscle microvascular blood flow, potentially contributing to acute hyperglycemia-induced insulin resistance [146].

Emerging research is also exploring the role of gut microbiota in nutrient flow and muscle health. Probiotics and prebiotics, which modulate the gut microbiota, are being investigated for their potential to improve nutrient absorption and muscle health [147].

Additionally, the concept of “nutritional periodization”, which involves adjusting nutrient intake to match the body’s needs at different times (such as pre- and post-exercise), is gaining attention as a strategy to maximize the anabolic response to nutrients [148,149].

### Summary of Evidence and Current Clinical Recommendations

Nutrient flow, which ensures the delivery of essential nutrients like amino acids and glucose to muscles, is critical for maintaining MPS. Impaired nutrient delivery, often due to endothelial dysfunction and insulin resistance, contributes to anabolic resistance. Nutrient flow is regulated by pathways such as mTORC and AMPK, with leucine and other essential amino acids playing crucial roles in activating MPS.

In clinical practice, optimizing nutrient flow through increased protein intake and the consumption of leucine-rich foods is key to improving MPS. Exercise, especially resistance training, helps enhance blood flow and nutrient delivery. Supplements like arginine, beetroot juice, and omega-3 fatty acids may further support nutrient flow by improving vascular function.

## 8. Anabolic Resistance and Anabolic Regulators in Sarcopenia

Anabolic resistance is a critical element in sarcopenia and is characterized by a diminished MPS response to anabolic stimuli such as nutrient ingestion and exercise [150]. This phenomenon is attributed, at least partly, to disruptions in the signaling pathways essential for muscle growth and repair, notably the mTORC pathway [150]. Research indicates that older adults show decreased phosphorylation of mTORC and its downstream targets in response to anabolic signals, leading to diminished MPS and impaired muscle mass maintenance [150]. 

Several pathophysiological mechanisms underpin anabolic resistance, including insulin resistance, chronic low-grade inflammation, and mitochondrial dysfunction [151]. Studies have identified insulin resistance and inflammation as major contributors to anabolic resistance, providing key therapeutic targets for improving muscle health. However, the complex interplay of these mechanisms implies that addressing one factor alone may not fully overcome anabolic resistance in older adults. Insulin resistance, prevalent among older adults, impairs the muscle’s ability to respond to anabolic signals, thereby decreasing MPS [151,152]. Chronic low-grade inflammation, often referred to as “inflammaging”, involves elevated levels of proinflammatory cytokines such as TNF-α and IL-6, which interfere with anabolic signaling and exacerbate muscle degradation [153]. Mitochondrial dysfunction contributes by reducing energy production and increasing oxidative stress, impairing muscle cell function and regeneration [154]. 

Addressing anabolic resistance involves multiple strategies, including optimizing protein intake, with greater protein quantities per meal necessary to effectively stimulate MPS in older individuals [155]. Optimizing protein intake, particularly leucine-rich protein, has consistently been shown to enhance MPS in older adults, helping counteract the effects of anabolic resistance. Supplementation with essential amino acids, particularly leucine, has shown promise in enhancing MPS due to its role in activating the mTORC pathway [155]. Nevertheless, anabolic resistance remains a significant barrier, and higher protein intakes are often necessary to achieve the desired results, which may be difficult for some older adults to sustain.

Pharmacological approaches include myostatin inhibitors [20], which are currently undergoing clinical trials, and selective androgen receptor modulators (SARMs) [85,156], which promote muscle growth without the adverse effects of traditional anabolic steroids. These pharmacological interventions, particularly SARMs, offer a potentially safer alternative to traditional anabolic therapies. However, both myostatin inhibitors and SARMs are still in experimental phases, and their long-term safety and efficacy in humans remain to be established. 

Resistance training is the most effective intervention for combating anabolic resistance, enhancing insulin sensitivity, reducing inflammation, and directly stimulating MPS through mechanical loading of the muscles [155]. Combining resistance exercise with protein supplementation can synergistically improve muscle mass and function [156]. 

Anti-inflammatory treatments, such as omega-3 fatty acids, are being explored for their potential to reduce systemic inflammation and improve muscle anabolic response [157]. Recently, the administration of an experimental drug targeting IL-11, a pro-inflammatory cytokine of the IL-6 family, increased muscle mass and strength in old mice, which was associated with up to 25% life extension [158]. The potential for anti-inflammatory therapies, particularly novel treatments targeting specific cytokines like IL-11, offers a promising approach to improving anabolic response and muscle mass in aging populations. Despite these exciting findings in preclinical models, human studies are still limited, and concerns about safety in long-term use remain. More research is needed before these treatments can be applied in clinical practice.

Hormonal therapies involving growth hormone (GH) and IGF-1 are also investigated for their anabolic effects, although they are not currently employed in clinical practice due to significant safety concerns, including the risk of cancer, diabetes, and other adverse effects, particularly in older adults [159].

### Summary of Evidence and Current Clinical Recommendations

Anabolic resistance refers to a diminished ability of muscle tissue to respond to anabolic stimuli, such as dietary protein and exercise. In older adults, anabolic resistance is a key contributor to the development and progression of sarcopenia. This resistance is largely mediated by impaired nutrient sensing, insulin resistance, and chronic low-grade inflammation, which reduce MPS. Key signaling pathways, such as mTORC1, play a central role in regulating MPS, and their activation can be compromised in the setting of anabolic resistance. The intake of leucine-rich proteins and the use of anabolic regulators, such as IGF-1 and testosterone, have shown potential in overcoming anabolic resistance.

To mitigate anabolic resistance in older adults, increasing protein intake is recommended, with a particular focus on leucine-rich sources, and distributing protein intake evenly across meals to enhance MPS throughout the day. Resistance training remains a highly effective strategy for improving anabolic sensitivity. In some cases, supplementation with anabolic regulators such as HMB may be considered. Nutritional strategies and exercise should be tailored to individual needs to optimize the anabolic response.

## 9. Neuromuscular Junction Transmission in Sarcopenia

In recent years, neuromuscular junction (NMJ) alterations have emerged as a key determinant in the pathogenesis of sarcopenia [160]. The NMJ is a complex structure involving presynaptic motor neuron terminals, the synaptic cleft, and postsynaptic muscle membrane. Efficient transmission at the NMJ is dependent on the release of the neurotransmitter acetylcholine (ACh) from synaptic vesicles in the presynaptic terminal. This process is tightly regulated by several proteins, including synaptotagmin, SNAP receptor (SNARE) complexes, and voltage-gated calcium channels. Upon release, ACh traverses the synaptic cleft and binds to nicotinic acetylcholine receptors (nAChRs) on the muscle membrane, leading to depolarization and subsequent muscle contraction [161].

**Figure 2:**
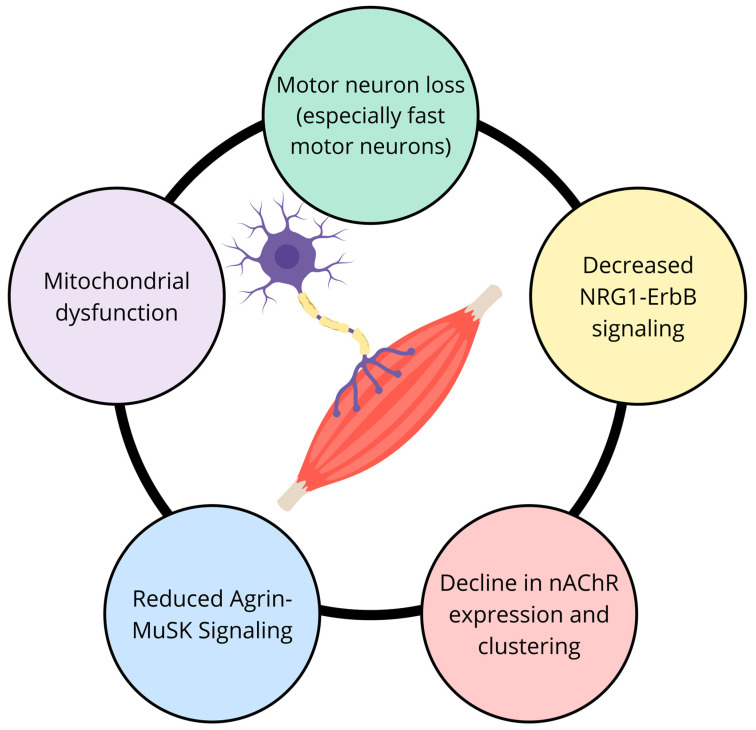
Age-related changes in neuromuscular junction potentially contributing to sarcopenia. Abbreviations: nAchR, nicotinic acetylcholine receptors; MuSK, muscle-specific kinase.

With aging, several alterations in NMJ structure and function contribute to sarcopenia (Figure 2). One significant change is the reduction in the number and function of motor neurons, leading to denervation of muscle fibers. This denervation results in the fragmentation and simplification of the NMJ, impairing efficient neurotransmission [160]. Additionally, advancing age is associated with a decline in the expression and clustering of nAChRs on the muscle membrane, further compromising synaptic efficacy [162]. Mitochondrial dysfunction within both neurons and muscle fibers also plays a role, as oxidative stress can damage NMJ components and exacerbate synaptic decline [160]. At the molecular level, several signaling pathways and molecules are implicated in the maintenance and degradation of the NMJ. The agrin-MuSK (muscle-specific kinase) pathway is critical for the formation and maintenance of NMJs. Agrin, released by motor neurons, activates MuSK on the muscle membrane, promoting the clustering of nAChRs and stabilizing the NMJ structure. With aging, there is a reduction in agrin levels and MuSK activity, leading to NMJ destabilization [163]. Another important pathway involves neuregulin-1 (NRG1) and its receptors, ErbB2/4, which are essential for the survival and function of motor neurons and muscle fibers. Decreased NRG1-ErbB signaling is associated with NMJ degeneration in aged muscles [164]. These findings provide insight into the molecular mechanisms underlying NMJ degeneration, offering promising therapeutic targets, such as agrin and MuSK signaling, that can potentially stabilize NMJs and improve muscle function in sarcopenia. However, the clinical translation of these molecular targets is still in its early stages, and more research is needed to evaluate their effectiveness in human populations.

To address sarcopenia through NMJ-targeted therapies, several strategies can be considered. One promising approach is the use of compounds that enhance NMJ stability and function. For example, acetylcholinesterase inhibitors, which prevent the breakdown of ACh in the synaptic cleft, can enhance neurotransmission and have shown potential in mitigating muscle weakness in sarcopenic mice [165]. Additionally, drugs that mimic or boost agrin and MuSK signaling could help maintain NMJ integrity [166]. Experimental therapies using recombinant agrin or small molecules that activate MuSK are currently under investigation [163,167]. These approaches show promise in preclinical models, particularly in improving NMJ function and muscle strength. However, their long-term safety and efficacy in humans have yet to be established, and they remain experimental.

In a recent study, Schellino et al. [168] tested the effects of ActR-Fc-nLG3, a protein that combines the soluble activin receptor, a potent myostatin inhibitor, with the *C*-terminal agrin nLG3 domain, in old mice. The authors found that this dual approach enhanced NMJ stability, improved muscle function, and increased overall endurance [168], suggesting a potential therapeutic strategy to counteract age-related muscle decline and sarcopenia. The dual action of myostatin inhibition and agrin enhancement represents an innovative approach that could offer synergistic benefits for NMJ and overall muscle health. However, this remains a preclinical finding, and further studies in humans are necessary to determine its therapeutic potential.

Gene therapy also holds potential for targeting NMJ degeneration. Delivering genes that encode for key NMJ proteins, such as agrin or NRG1, directly to muscle tissues using viral vectors could restore normal NMJ function [169,170]. This approach could provide long-term benefits by addressing the underlying molecular deficits in sarcopenia. However, gene therapy is still in its early stages, and concerns about safety, delivery, and cost remain significant barriers to clinical application.

Another area of intervention is the modulation of oxidative stress and mitochondrial function. Antioxidants and compounds that improve mitochondrial health may protect NMJ components from age-related damage. For example, mitochondrial-targeted antioxidants such as MitoQ have shown promise in both preclinical models of sarcopenia and humans [171,172]. Enhancing mitochondrial biogenesis by activating pathways such as PGC-1α may also support NMJ maintenance [173,174]. Targeting mitochondrial dysfunction through antioxidants and PGC-1α activation holds promise for preventing NMJ decline and improving muscle endurance. However, the long-term efficacy of these interventions, particularly in reversing established NMJ degeneration, requires further investigation.

Physical activity and exercise remain the most effective non-pharmacological interventions for sarcopenia. Physical activity stimulates NMJ remodeling and enhances synaptic transmission efficiency [175,176]. Resistance training, in particular, has been shown to increase the expression of NMJ-related genes and improve neuromuscular function in older adults [73]. Combining exercise with pharmacological or gene-based therapies could synergistically enhance their effectiveness.

Emerging research is also exploring the role of exosomes, small extracellular vesicles that mediate intercellular communication, in NMJ maintenance [177,178]. Exosomes derived from stem cells or young muscle tissues contain factors that can rejuvenate aged NMJs [179]. Developing exosome-based therapies could offer a novel approach for treating sarcopenia by delivering bioactive molecules that promote NMJ repair and regeneration [180]. Exosome-based therapies offer a novel and potentially less invasive approach to NMJ repair, leveraging the natural regenerative properties of stem cells. However, this approach is still in its experimental phase, and further research is needed to optimize the use of exosomes in clinical applications.

### Summary of Evidence and Current Clinical Recommendations

With aging, NMJ transmission efficiency declines, contributing to muscle weakness and sarcopenia. Structural and functional alterations in the NMJ impair signal transmission, leading to decreased muscle strength. This degeneration is associated with both aging and disuse, with chronic inflammation and oxidative stress playing significant roles. Strategies to preserve NMJ integrity, such as exercise, nutritional support, and neuromodulatory agents, are being investigated.

Exercise, particularly resistance and neuromuscular training, is recommended to maintain or restore NMJ function. Nutritional interventions that include omega-3 fatty acids and antioxidants may help preserve NMJ integrity by reducing inflammation and oxidative stress. In some cases, pharmacological agents that enhance acetylcholine receptor function or protect NMJ transmission may be considered, although more research is needed to confirm their clinical utility.

## 10. Conclusions

Sarcopenia is a complex and multifactorial clinical condition in which multiple determinants may have a different weight from person to person [181]. As a result, each individual with sarcopenia may have a unique substrate on which to intervene and this does not only depend on the features of this disease, but also on the great variability in terms of health status and functional capacities within the population of older adults and the coexistence of chronic diseases and geriatric syndromes [182,183,184]. Regarding the long-standing issue of the definition of sarcopenia, we are at a point where the main diagnostic algorithms have been developed, with the next steps being to further refine current definitions and diagnostic workups, as well as the relationships linking it to other conditions resulting from changes in body composition, such as osteoporosis (osteosarcopenia) and obesity (sarcopenic obesity) [185]. Nutritional interventions and exercise have been established as key treatments for sarcopenia. Emerging therapeutic strategies, including pharmacological interventions, gene therapy, and mitochondrial-targeted antioxidants, offer promising avenues for mitigating sarcopenia (Table 3). However, more research is needed to validate the efficacy and safety of these therapies in clinical trials and real-world settings, particularly in older adults with multiple comorbidities. The combination of conventional interventions with pharmacological or gene-based therapies appears particularly promising for enhancing muscle function and NMJ stability. Yet, further clinical trials are essential to explore how these combinations can be optimized for long-term outcomes and to address concerns about potential side-effects. Exosome-based therapies and the modulation of oxidative stress and mitochondrial function are intriguing areas of ongoing research. While early studies have shown potential, more rigorous, long-term studies are required to establish their effectiveness and determine their role in standard sarcopenia treatment protocols. Addressing the molecular deficits underlying sarcopenia through these innovative approaches holds potential for significantly improving the quality of life and physical function in the aging population. However, it is essential to focus on addressing the existing research gaps, particularly by conducting long-term clinical trials, real-life cohort studies, and advancing translational research. This will be crucial for gaining a deeper understanding of the long-term efficacy, safety, and practical applicability of these emerging therapies in clinical settings.

## Figures and Tables

**Table 1:** Nutritional strategies for sarcopenia and their effects on muscle health.

Nutritional Intervention	Key Nutrients/ Components	Effects on MPS	Effects on Inflammation	Effects on Muscle Regeneration	References
Protein intake	High-quality protein (e.g., whey, casein)	Stimulates MPS, essential for muscle repair and growth	May reduce inflammation when combined with exercise	Supports overall muscle regeneration	[49,50]
BCAA supplementation	BCAA, particularly leucine	Stimulates MPS via the mTORC1 pathway	Reduces markers of inflammation	Enhances satellite cell activation and muscle repair	[50,51]
Omega-3 fatty acids	EPA, DHA	Modulate MPS, especially in response to resistance training	Strong anti-inflammatory effects, reduce cytokine levels	Enhance muscle regeneration through improved mitochondrial function	[52,53]
HMB	Metabolite of leucine	Promotes MPS, decreases muscle protein breakdown	Anti-inflammatory effects by inhibiting NF-κB signaling	Enhances muscle mass and function in older adults	[54,55]
Creatine	Creatine monohydrate	Supports rapid ATP production, increases training capacity	Indirectly reduces inflammation by improving muscle bioenergetics	Enhances satellite cell proliferation and muscle fiber repair	[56,57]
Arginine	Amino acid, precursor of NO	Promotes MPS via the mTORC1 pathway	Vasodilatory and anti-inflammatory effects via increased NO production	Improves blood flow to muscles, supporting regeneration and recovery	[58,59]
Vitamin D	Cholecalciferol, ergocalciferol	Enhances MPS, especially in individuals with low baseline levels	Modulates immune response, potentially reducing inflammation	Supports muscle cell growth and differentiation	[49,60]
Antioxidants	Vitamin C, Vitamin E, Coenzyme Q10	May support MPS indirectly by reducing oxidative damage to muscle cells	Reduce oxidative stress and inflammation	Protect muscle cells from oxidative damage, supports recovery	[52,61]
Dietary nitrates	Beetroot juice, spinach, arugula	Improve mitochondrial efficiency, indirectly supporting MPS	Reduce blood pressure and inflammation	Enhance blood flow to muscles, supports muscle endurance	[62,63]

Abbreviations: ATP, adenosine triphosphate; BCAA, branched-chain amino acid; DHA, docosahexaenoic acid; EPA, eicosapentaenoic acid; HMB, β-hydroxy β-methylbutyrate; MPS, muscle protein synthesis; mTORC1, mechanistic target of rapamycin complex 1; NF-κB, nuclear factor kappa-light-chain-enhancer of activated B cells; NO, nitric oxide.

**Table 3:** Emerging pharmacological interventions for sarcopenia.

Pharmacological Intervention	Mechanism(s) of Action	Potential Benefits	Challenges/ Side-Effects	References
Myostatin inhibitors	Block myostatin signaling, which inhibits muscle growth	Increase muscle mass and strength	Inconsistent results in improving muscle function and reducing physical frailty in clinical trials. Significant side-effects.	[19,25]
Anti-inflammatory therapies	Reduce chronic inflammation which impairs muscle regeneration	Potentially enhance muscle regeneration and reduce muscle atrophy	Significant side-effects, especially in older adults (e.g., NSAIDs),	[30,157]
Stem cell therapy	Promotes muscle regeneration by leveraging stem cell populations	Enhances muscle repair and regeneration through cell-based therapies	Promising results from clinical trials; further data on efficacy and safety are needed.	[44,46]
Gene editing (CRISPR/Cas9)	Targets genetic pathways to enhance muscle growth and repair	Potential to correct genetic mutations and increase muscle mass	Ethical considerations and early-stage clinical applications.	[42,43]
Mitochondrial enhancers	Improve mitochondrial biogenesis and function	Enhance muscle energy metabolism and reduce muscle fatigue	Require further validation in clinical settings.	[61,78]
Selective androgen receptor modulators	Promote muscle growth without the adverse effects of anabolic steroids	Increase muscle mass and strength	Potential risks and long-term safety concerns.	[85,156]
Acetylcholinesterase inhibitors	Prevent the breakdown of acetylcholine, enhancing neuromuscular junction function	Mitigate muscle weakness associated with sarcopenia	Side-effects related to acetylcholine metabolism.	[165,168]
MicroRNA modulation	Modulates gene expression to enhance muscle regeneration	Novel approach to target specific pathways involved in muscle regeneration	Early stage of research; potential off-target effects.	[43,48]
Exosome-based therapies	Utilize small extracellular vesicles for targeted delivery of bioactive molecules	Promote NMJ repair and muscle regeneration	Still in early research stages; potential delivery challenges.	[177,180]

Abbreviations: CRISPR, Clustered Regularly Interspaced Short Palindromic Repeats, NMJ, neuromuscular junction; NSAIDs, non-steroidal anti-inflammatory drugs.

## Data Availability

Data sharing is not applicable to this article as no new data were created or analyzed in this study.
